# Electronic Health Record Data Collection Practices to Advance Standardization and Interoperability of Patient Preferences for Interpretation Services: Qualitative Study

**DOI:** 10.2196/62670

**Published:** 2025-01-31

**Authors:** Krysta Heaney-Huls, Rida Shams, Ruth Nwefo, Rachel Kane, Janna Gordon, Alison M Laffan, Scott Stare, Prashila Dullabh

**Affiliations:** 1 NORC at the University of Chicago Chicago, IL United States; 2 Yale School of Public Health New Haven, CT United States; 3 Riverbend Health Psychology, PLLC Salt Lake City, UT United States; 4 Office of Minority Health, Centers for Medicare & Medicaid Services Baltimore, MD United States

**Keywords:** health information exchange, interoperability, electronic health records, interpreter, limited English proficiency, communication barriers

## Abstract

**Background:**

Poor health outcomes are well documented among patients with a non-English language preference (NELP). The use of interpreters can improve the quality of care for patients with NELP. Despite a growing and unmet need for interpretation services in the US health care system, rates of interpreter use in the care setting are consistently low. Standardized collection and exchange of patient interpretation needs can improve access to appropriate language assistance services.

**Objective:**

This study aims to examine current practices for collecting, documenting, and exchanging information on a patient’s self-reported preference for an interpreter in the electronic health record (EHR) and the implementation maturity and adoption level of available data standards. The paper identifies standards implementation; data collection workflows; use cases for collecting, documenting, and exchanging information on a patient’s self-reported preference for an interpreter; challenges to data collection and use; and opportunities to advance standardization of the interpreter needed data element to facilitate patient-centered care.

**Methods:**

We conducted a narrative review to describe the availability of terminology standards to facilitate health care organization documentation of a patient’s self-reported preference for an interpreter in the EHR. Key informant discussions with EHR developers, health systems, clinicians, a practice-based research organization, a national standards collaborative, a professional health care association, and Federal agency representatives filled in gaps from the narrative review.

**Results:**

The findings indicate that health care organizations value standardized collection and exchange of patient language assistance service needs and preferences. Informants identified three use cases for collecting, documenting, and exchanging information on a patient’s self-reported preference for an interpreter, which are (1) person-centered care, (2) transitions of care, and (3) health care administration. The discussions revealed that EHR developers provide a data field for documenting interpreter needed data, which are routinely collected across health care organizations through commonly used data collection workflows. However, this data element is not mapped to standard terminologies, such as Logical Observation Identifiers Names and Codes (LOINC) or Systematized Medical Nomenclature for Medicine–Clinical Terminology (SNOMED-CT), consequently limiting the opportunities to electronically share these data between health systems and community-based organizations. The narrative review and key informant discussions identified three potential challenges to using information on a patient’s self-reported preference for an interpreter for person-centered care and quality improvement, which are (1) lack of adoption of available data standards, (2) limited electronic exchange, and (3) patient mistrust.

**Conclusions:**

Collecting and documenting patient’s self-reported interpreter preferences can improve the quality of services provided, patient care experiences, and equitable health care delivery without invoking a significant burden on the health care system. Although there is routine collection and documentation of patient interpretation needs, the lack of standardization limits the exchange of this information among health care and community-based organizations.

## Introduction

### Background

Health disparities are a prevalent issue in health care delivery in the United States today. Among the contributors to care inequities are barriers to language assistance services. Barriers to language assistance services are associated with misdiagnosis and inappropriate treatment by providers [1] and contribute to poor health outcomes that are well documented among patients with a non-English language preference (NELP) [2]. Compared with English-speaking patients, patients with NELP experience worse health outcomes, such as undiagnosed or uncontrolled hypertension, prolonged hospital stays, and poor asthma control [3]. Among Hispanic communities, which represent the fastest growing non-English language population in the United States, stroke is a leading cause of death and has been attributed to factors such as barriers to language assistance services [4]. Studies have also found that patients who experience barriers to language assistance services have a reduced likelihood of physician and mental health provider visits and are more likely to have an unplanned emergency department visit compared with patients who are proficient in English. Furthermore, patients with NELP are at an increased risk for unplanned repeat emergency department visits within 72 hours of discharge [5,6].

 Interpretation services represent both a growing and unmet need in the United States health care system [7]. The National Council on Interpreting in Health Care defines a medical interpreter as an individual who “interprets in health care settings of any sort, including doctor’s offices, clinics, hospitals, home health visits, mental health clinics, and public health presentations” [8]. In 2021, the Migration Policy Institute estimated that approximately 26 million individuals in the United States reported a NELP [9,10].

**Figure 1:**
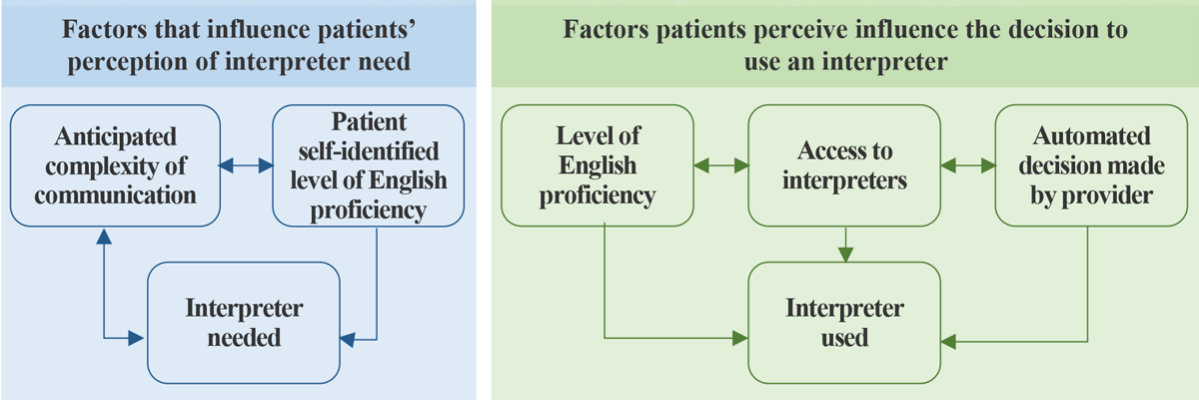
Factors that patients say influence whether an interpreter is needed or used (adapted from Schwei et al [12]).

The use of interpreters has been found to improve the quality of care for patients with NELP, with patients reporting overall high satisfaction with care and communication with their care team [11]. However, despite the benefits, rates of interpreter use are consistently low, and additional research is needed to better understand how patients assess the need for and use an interpreter [12]. The findings from Schwei et al [12] inform our current understanding of a patient’s decision-making processes (Figure 1) and could be beneficial to inform how health care organizations engage with patients to gather and use data on a patient’s self-reported preference for an interpreter.

In the absence of medical interpreters, patients with NELP are left to rely on ad hoc interpreters such as family members, hospital staff members, or their own language fluency to obtain needed medical information. This leaves room for a variety of common interpretation errors to occur such as omissions, embellishments, false fluency, paraphrasing, and family members or ad hoc interpreters providing their own opinions [13]. Interpreter use can also be impacted by concerns from health providers or patients regarding the privacy and confidentiality of the communications between the patient and interpreter [5,14,15]. Patients may be reluctant to use an interpreter if they are uncertain of the privacy and confidentiality protections. These issues highlight the importance of both identifying patient language access needs and providing linguistically appropriate interpretation and translation services.

The standardized collection and exchange of patient interpretation needs can help improve access to appropriate language assistance services. Once collected from patients, this data can be stored in the electronic health record (EHR) and accessed by the patient’s care team to support person-centered care or exchanged electronically with other providers (eg, specialists and long-term care facilities) to support transitions of care.

### Objectives

This paper examines current practices for collecting, documenting, and exchanging information on a patient’s self-reported preference for an interpreter in the EHR. The paper identifies data standards implementation, data collection workflows, use cases for information on a patient’s self-reported preference for an interpreter, challenges to data collection and use, and opportunities to advance the standardized collection and use of the interpreter needed data element to facilitate patient-centered care.

## Methods

We used 2 methods to assess the collection, documentation, and use of information on a patient’s self-reported preference for an interpreter, which are (1) a narrative review and (2) key informant discussions.

### Literature Scan

We conducted a narrative review [16] to describe the availability of terminology standards to facilitate health care organization documentation of a patient’s self-reported preference for an interpreter in the EHR, and the implementation maturity and adoption level of available data standards.

We searched both the peer-reviewed and gray literature using PubMed and Google (Textbox 1). We also searched the websites of standards development organizations including the Health Level Seven (HL7) website and Confluence pages, the Assistant Secretary for Technology Policy/Office of the National Coordinator for Health Information Technology (ASTP/ONC) website, the Logical Observation Identifiers Names and Codes (LOINC) website, the Gravity Project website, PCORnet (the National Patient Centered Clinical Research Network), and the Centers for Medicare & Medicaid Services (CMS) website. We conducted supplemental searches on barriers to language assistance services and available standards (eg, Systematized Medical Nomenclature for Medicine-Clinical Terminology [SNOMED-CT] and Clinical Document Architecture) based on discussions with key informants. In total, we included a total of 31 sources.

**Example search terms.**

**PubMed search terms**

- “interpreter needed” AND “data element”, “interpreter” AND “data element”, “interpreter” AND “data” AND “EHR”, language translator needed data element, interpretation, communication barriers, limited English proficiency, and “translating*”

**Gray literature keywords**

- “interpreter*”, “interpreter needed”, “interpreter service”, “interpreter required”, “translating*”, “language translator”, “communication facilitator*”, United States Core Data for Interoperability (USCDI), “data element”, electronic health record (EHR), language translator needed data element, inclusion of language preference in EHR, inclusion of language preference in EHR, Centers for Medicaid & Medicaid Services (CMS) Data Element Library (DEL) Health Information Technology Workgroup, Health Level 7 (HL7) Workgroup, Office of the National Coordinator (ONC), Gravity Project, and PCORnet Common Data Model.

### Key Informant Discussions

We conducted key informant discussions to complement findings from the literature scan and contextual information gaps by identifying emergent topics not yet reflected in the literature. We identified a purposive sample of informants across 3 stakeholder categories to gather diverse perspectives on the collection and use of data on a patient’s self-reported preference for an interpreter in the EHR, which are (1) EHR developers (n=3), (2) health systems and clinicians (n=8), and (3) policy-making and advocacy organizations (n=5). The EHR developers included in the study represented over 70% of the market share by bed count in the inpatient setting [17] and nearly half of the ambulatory market share among clinicians that report using a 2015 edition certified EHR product [18]. Health system and clinician informants included representatives from academic medical centers, a Federally Qualified Health Center, a primary care clinic, an integrated health care system, and acute care hospitals. Finally, policy-making and advocacy organization informants were comprised of Federal agency staff, language service professionals, members of a national consensus–based standards collaborative, and a community health center association. The key informants provided perspectives on the experience of a wide variety of health care organizations’ processes for collecting, documenting, and using information on a patient’s self-reported preference for an interpreter.

We generated transcripts for each key informant discussion. We used qualitative content analysis to identify key themes using the existing United States Core Data for Interoperability (USCDI) ONC New Data Element and Class Submission System, focusing on new data element submission requirements and emergent themes from gaps in the literature. The USCDI ONC New Data Element and Class submission requirements include (1) similar or related data elements in USCDI, (2) why the new data element should be considered separately, (3) main use cases to support the adoption of the data element into the USCDI, (4) estimates of the breadth of applicability of the use cases for the new data element, (5) data element maturity (eg, existing vocabulary, terminology, or content standards), (6) availability of additional technical specifications, (7) level determination representing the use of the new data element, (8) level of electronic exchange with external organizations, and (9) challenges to implementation (eg, restrictions on standardization or use, privacy and security concerns, and implementation burden) [19].

### Analytic Approach

The findings from the literature scan were summarized and grouped into two primary categories, which are (1) standards for certified EHR and health information technology systems and (2) health care quality improvement. We used a data abstraction matrix to identify themes from the key informant discussions. We abstracted data from the discussions into five domains: (1) current practices for collecting information on a patient’s self-reported preference for an interpreter, (2) use cases for this information, (3) EHR product support for an interpreter needed data element, (4) interoperability standards, and (5) challenges to collecting, documenting, and using information on a patient’s self-reported preference for an interpreter. The authors also identified common themes from across the 5 domains to identify key considerations and opportunities to improve data interoperability and expand upon the use of the interpreter needed data element.

### Ethical Considerations

This study was reviewed by the NORC at the University of Chicago Institutional Review Board and did not meet the definition of research with human subjects set forth in Federal Regulations at 45 CFR 46.102.

## Results

### Overview

Health care organizations routinely collect information on a patient’s self-reported preference for an interpreter and preferred language data to facilitate interpretation service delivery. Health care organizations recognize the limitation of using either preferred language or English proficiency alone to identify a patient’s preference or need for an interpreter and, therefore, collect a patient’s self-reported preference for interpretation services in tandem with preferred language to accurately reflect language service access needs.

### Current Health Care Organization Data Collection Practices

We did not find evidence of health care organizations using available standard terminologies (eg, LOINC and SNOMED-CT) in the literature. Key informant interviews with health systems, clinicians, and EHR developers affirmed this finding, stating that health care organizations use structured fields to document a patient’s self-reported preference for an interpreter in the EHR but do not map this data to available clinical terminologies.

**Figure 2:**
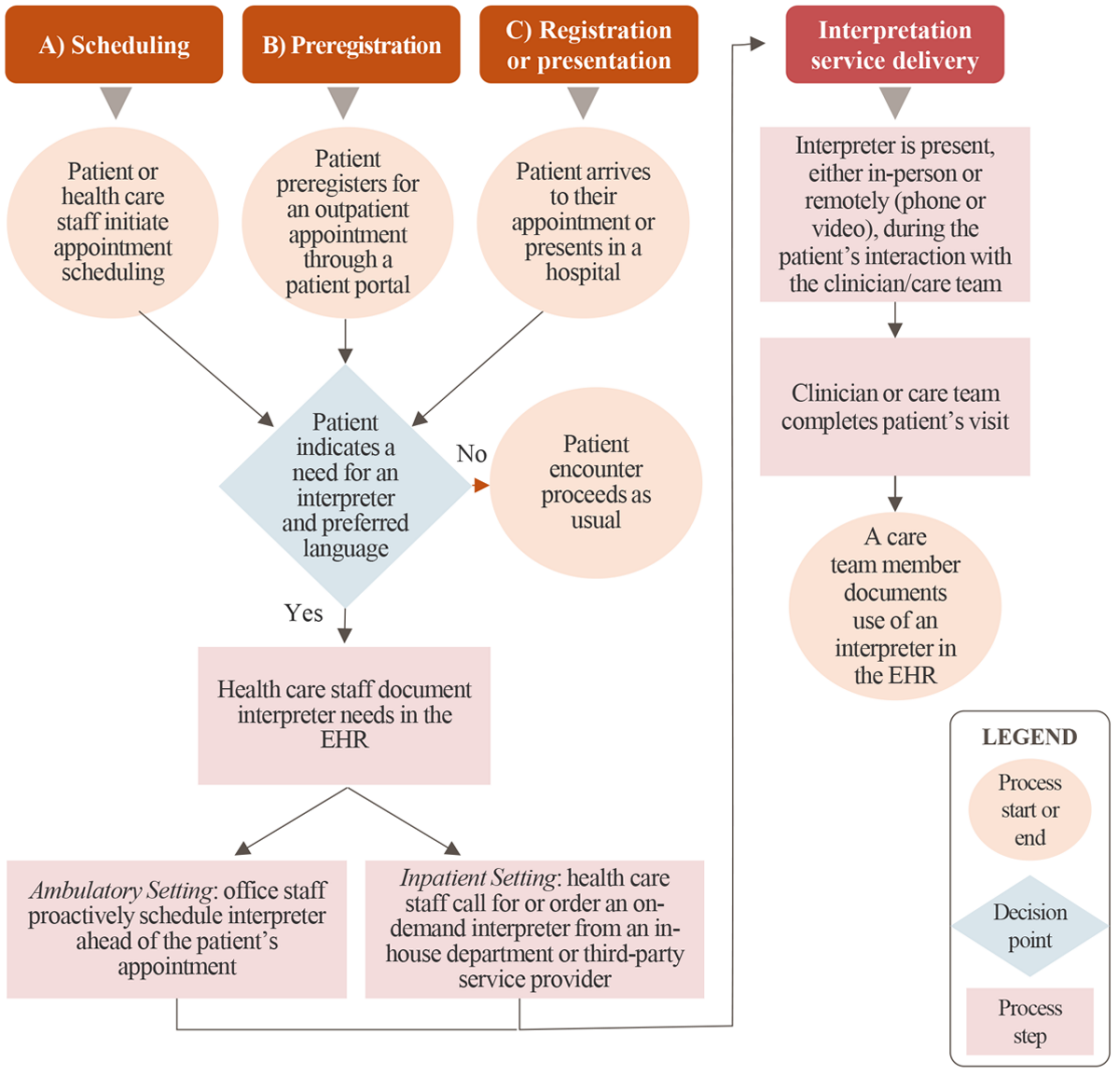
Interpretation service requests and delivery workflow. EHR: electronic health record.

Through the key informant discussions, we identified common practices for the collection, documentation, and use of information on a patient’s self-reported preference for an interpreter in both the ambulatory and inpatient settings. We present a workflow diagram for the common data collection and documentation processes in Figure 2 and describe each step below.

In Figure 2, three events (A-C) begin the process of gathering information from the patient on their self-reported preference for an interpreter and the subsequent provision of interpretation services. However, nuances exist depending on the EHR, organizational practices, care delivery setting, the availability of interpretation services, and interpretation service delivery method. Event A indicates scheduling a patient encounter. When a patient or health care staff initiates appointment scheduling, office staff members document the need for interpretation services in the EHR through an interpreter needed data field. Office staff members may proactively schedule interpretation services for the patient. Event B begins with patient preregistration. During preregistration, the patient may self-report their preference for an interpreter and their preferred language within the patient portal. Office staff members document this information in the patient’s record and may proactively schedule interpretation services for the patient. Event C occurs with patient registration or when a patient presents in a hospital. When a patient self-reports their preference for an interpreter at arrival for an outpatient appointment or presents at a hospital, staff members document this information in the EHR and contact an interpreter for on-demand service.

Interpretation services can be delivered in-person or remotely through phone or video connection. Providers may use an interpreter from an internal interpretation services department, or a third-party vendor, or rely upon a clinician or staff member who is medically proficient in the patient’s preferred language. Outpatient clinics often proactively schedule in-person or remote interpreters from third-party services because they may have collected a patient’s self-reported preference for an interpreter before the patient encounter. If the clinic serves a significant number of patients with NELP, they may have in-house interpreters available or staff members who are medically proficient (ie, someone whose non-English language skills include communicating medical terminology and information) in the patient’s preferred language. In the inpatient setting, interpretation services are provided either on-demand from an in-house interpretation service department or remotely (eg, through a video connection). Upon completion of the patient visit, a member of the care team may document the use of the interpreter within the patient notes section of the EHR.

### Use Cases for Data on a Patient’s Self-Reported Preference for an Interpreter

#### Overview

Using the information on a patient’s self-reported preference for an interpreter can assist health care organizations in providing more appropriate patient-centered care and eliminate guesswork or assumptions made by health care teams regarding which patients need an interpreter, ultimately improving the patient experience. Key informants described three primary use cases for the collection, documentation, and use of information on a patient’s self-reported preference for an interpreter, which are (1) person-centered care, (2) transitions of care, and (3) health care administration (Table 1).

**Table 1:** Use cases and illustrative quotes for using data on a patient’s self-reported preference for an interpreter.

Use case	Illustrative key informant quotes
Person-centered care	The biggest benefit of collecting interpreters needed is improving the patient care experience for patients with limited English proficiency. [Health system representative] Accuracy of language is especially important because you need to understand what specific problems the patient is experiencing and ask follow-up questions to make a solid differential diagnosis. [FQHC^a^ representative] It helps drastically with patient satisfaction when we think about being able to communicate with your provider, understand what they’re saying, and feeling comfortable that you are understanding medical jargon in your native tongue. [Health system representative]
Transition of care	If that information is exchanged, then it can be good, because other providers have that information in advance, and can arrange for interpretation services. This could be really good in situations where it’s a referral. [Health system representative] I think one place where exchanging this field would be helpful is when we bring in contracted specialists...So, if a patient was going for a first-time visit with an external provider, that would be valuable for the specialist to know, and to meet with patient needs. [Health system representative] I could see exchanging Interpreter Needed being useful for patients that are being transferred to long-term acute care hospitals (LTACH). That facility should have the ability to see what language the patient needs to be served in. [FQHC representative]
Health care administration	Requiring the [interpreter needed] data element might help normalize requesting interpreter services, and/or create a downstream effect of improving the interpreter services payment structure. [Health system representative]

^a^FQHC: Federally Qualified Health Center.

#### Use Case 1: Person-Centered Care

Improving communication with patients and ensuring person-centered care is the central purpose behind collecting information on a patient’s self-reported preference for an interpreter and their preferred language. Key informants and available literature both indicate that interpretation services improve patient comprehension of clinician discussions, patient care quality, and care processes [11,20].

Patients with NELP who receive professional interpretation services are less likely to be readmitted to the hospital and more likely to experience a shorter length of stay than patients with NELP who do not receive interpretation services [11]. Key informants echoed that interpretation services can enhance the delivery of safer patient care and patient decision-making by facilitating better communication between providers and patients regarding patient preferences, medical and nonmedical care needs, clinical information, and care instructions [21].

Finally, collecting information on a patient’s self-reported preference for an interpreter also facilitates services for patients with communication needs beyond spoken language, such as indications for an American Sign Language interpreter.

#### Use Case 2: Transitions of Care

Multiple key informants stated that exchanging data on a patient’s interpretation needs facilitates care transitions and referrals to specialty care, social care organizations, and postacute care (PAC) facilities. Exchanging information on a patient’s self-reported preference for an interpreter allows specialists to arrange interpretation services in advance of the patient’s appointment and may decrease the likelihood that patients are lost to follow-up due to interpretation service scheduling issues. Sharing patient language assistance needs through social care coordination platforms such as Unite Us facilitates patient access to interpreters at community partner organizations.

PAC facilities are required to collect data on whether a patient needs or wants an interpreter using standardized patient assessments under the CMS Quality Reporting Program. The data are collected using the Interpreter Needed LOINC terminology [22], allowing for interoperable data exchange among postacute providers and timely coordination of interpretation services.

#### Use Case 3: Health Care Administration

A few key informants noted that collecting data on the number of patients requiring interpretation services assists health care administrators in tailoring such services to adequately serve their patient population. For example, administrators can provide in-person interpretation services for the most requested languages and offer telephonic or remote interpretation services for less commonly requested languages.

Administrators also use information on a patient’s self-reported preference for an interpreter to fulfill reporting requirements and support quality improvement efforts. For example, Federally Qualified Health Centers are required to report the number of patients “best served in another language” as part of the Health Resources and Services Administration Uniform Data System. In addition, one of the 2023 quality improvement activities for the CMS Quality Payment Programs is to “Create and Implement a Language Access Plan” that adheres to the National Standards for Culturally and Linguistically Appropriate Services in Health and Health Care [23,24].

### EHR Products Support Documentation of Data on a Patient’s Self-Reported Preference for an Interpreter

Key informants from the 3 EHR developers stated that functionality to document information on a patient’s self-reported preference for an interpreter is available to health care organizations through a structured field in the EHR.

### Interpreter Needed Data Element Interoperability Standards

There are well-specified standard terminologies for the interpreter needed data element. The literature and key informants identified both LOINC and SNOMED-CT codes for identifying a patient’s self-reported preference for an interpreter: LOINC code 54588-9, “patient/resident’s need or want an interpreter to communicate with a doctor or health care staff” and the SNOMED-CT code 315593009, “need for interpreter (finding)” [25,26]. The ASTP/ONC 2024 Interoperability Standards Advisory identifies LOINC code 54588-9 as an available value set to use when communicating information in a patient’s preferred language [27].

Mapping information on a patient’s self-reported preference for an interpreter to one or both standards can facilitate the exchange of these data between providers, community-based organizations, and others to support patient needs. The HL7 Fast Healthcare Interoperability Resources (FHIR) and Clinical Document Architecture standards define how health care data can be exchanged between health care organizations with different health IT systems [28,29]. In addition, informants identified a FHIR standard (the FHIR Patient Resource-Interpreter Required) for exchanging information on whether a patient “requires an interpreter to communicate health care information to the practitioner” along with patient demographic and administrative data. This FHIR standard offers a practical solution to exchanging these data [30,31]. While our key informants reported that health care organizations do not routinely map information on a patient’s self-reported preference for an interpreter to standard terminology, representatives from the 3 EHR developers we spoke to indicate that mapping can be implemented with minimal burden.

### Challenges

Our key informants revealed the following 3 potential challenges to using information on a patient’s self-reported preference for an interpreter for person-centered care and quality improvement, which are (1) lack of adoption of available data standards, (2) limited electronic exchange, and (3) patient mistrust (Table 2).

**Table 2:** Challenges to using information on a patient’s self-reported preference for an interpreter and illustrative key informant quotes.

Challenge	Illustrative key informant quotes
Lack of adoption of available data standards	I don’t think Interpreter Needed is exchanged right now in a structured field...When I do see data coming through from other systems, I see interpreter information coming through in a free text field. [Health system representative] If it’s a continuation of care document (CCD) that we’re sending, it’s not a required field. I did check with our [EHR^a^ developer lead], and we aren’t sharing that Interpreter Needed field. It’s currently not technically possible...I imagine if it was something required by regulators, that might spur development. [Health system representative]
Limited electronic exchange of interpreter needed data	When we refer patients to specialists, the need for an interpreter is often included in free text notes and tends to get lost. I know anecdotally of instances where the specialist’s office misses that note, so they don’t know to provide an interpreter for the patient. Because it’s so challenging for patients to make those appointments in the first place, if the interpreter is not available at the specialist’s office the first time, it is common for them to never reschedule. [FQHC^b^ representative] I would guess that Interpreter Needed is a bit difficult to exchange between healthcare systems because it is not consistently labeled with one code” [FQHC representative]
Patient mistrust	This [interpreter needed] standardized data could also be used in a discriminatory way. On one hand, I want to advocate for patients to have better access to interpreters, but I also don’t want it to be used by providers to avoid patients that do need an interpreter. We need to be careful about how we use that information. [Health system representative] Another issue is that it can feel like there will be an added charge or cost to you if you request a translator, even if it is a free service. Patients don’t always feel like they’re entitled to ask for those services. Even with Public Charges, when they’re told they’re entitled to interpretation services, they may not feel like they can access those resources. [Language services professional representative]

^a^EHR: electronic health record.

^b^FQHC: Federally Qualified Health Center.

### Lack of Adoption of Available Data Standards

Key informants noted that while most health care organizations currently collect interpreter needed data, most do not map this data to an existing terminology standard. Limited mapping impedes data interoperability and efficient monitoring, tracking, and reporting on the need for interpretation services.

### Limited Electronic Exchange of Interpreter Needed Data

Due to the lack of data standardization, there is limited electronic exchange of interpreter needed data among most health care organizations. Key informants stated that a patient’s self-reported preference for an interpreter is often exchanged as free text in an administrative or clinical note, or it is shared through phone or fax when notifying a provider that a patient has requested an interpreter. Without standardized electronic exchange, communication of patient needs and preferences is inefficient, creating additional burden on the patient and room for care coordination errors. For instance, key informants shared examples of clinical notes indicating a patient’s self-reported preference for an interpreter not being reviewed, causing delayed or missed care if an interpreter is not available at the specialist’s office when the patient arrives for the appointment.

### Patient Mistrust

The findings from the literature revealed that collecting a patient’s self-reported preference for an interpreter from all patients may pose implications for patient trust. For example, a patient with NELP who would benefit from interpretation services may decline due to concerns about privacy and stigmatization [32]. Similarly, negative past experiences with interpreters (eg, misinterpretations or extended wait times for interpretation services) may lead to a patient declining interpretation service [33]. A few key informants also suggested that patients with NELP may decline an interpreter because they believe they will be billed for using those services, even when the patient is informed that they will not be charged. Although these findings transcend standardized collection and use of interpreter needed data, they are important challenges for health care organizations to consider when asking patients for this information.

## Discussion

### Principal Findings

Overall, while EHR developers and health systems indicated that interpreter needed data are routinely collected and used by most health care organizations, these data are not often mapped to available standards within the EHR. All the health care organizations and EHR developers we spoke with described well-defined administrative and clinical workflows to facilitate data collection of patients’ self-reported preference for an interpreter.

#### Collection of Interpreter Needed Data Can Improve Quality of Care

Despite limited standardization, our findings suggest the collection, documentation, and use of interpreter needed data can improve the quality of care for patients with NELP. The provision of language and communication services for individuals with a NELP or other communication needs (eg, those who are deaf or hard of hearing) can facilitate the accurate exchange of information regarding prevention, symptoms, diagnosis, treatment, care coordination, discharge planning, and shared decision-making between patients and their care team [34,35]. In short, addressing communication needs may assist in improving patient satisfaction, patient safety, and health outcomes of historically underserved populations.

#### Use of Interpreter Needed Data Has the Potential to Enhance Health Equity Research

Much of the research on English proficiency–related disparities in health care uses preferred language data abstracted from the EHR, patient experiences of care surveys (eg, Consumer Assessment of Healthcare Providers and Systems), and standardized PAC patient assessments to identify care gaps. Access to more accurate data on interpretation needs could assist researchers in more granularly identifying important health disparities among patients with NELP, understanding experiences of care among NELP individuals, and longitudinally tracking disparities. In addition, the use of standardized interpreter needed data alongside preferred language can support the development of more accurate estimates of language assistance needs that can be used to define quality measures for language service access for patients with NELP and help providers understand ways to better serve patients with NELP.

#### Greater Adoption of Standardized Interpreter Needed Data Is Necessary

The National Standards for Culturally and Linguistically Appropriate Services in Health and Health Care provide implementation guidance for offering communication and language assistance, which includes developing processes for identifying the languages an individual communicates in and documenting this information in the patient’s medical record [34]. Our findings support using available LOINC and SNOMED-CT codes for documenting a patient’s self-reported preference for an interpreter. Adoption of these standards will further efforts to use interpreter needed data for quality improvement initiatives aimed at achieving the Quintuple Aim.

#### Exchange of Interpreter Needed Data Has the Potential to Improve Disease Surveillance

EHR developers and health system key informants noted the potential benefits of exchanging interpreter needed data between health care organizations and public health authorities for the purpose of certain mandated public reporting, such as disease surveillance. For example, contact tracing could be more effective if information on individuals with language service access needs was shared in advance with public health authorities, thus improving communication regarding disease risk and transmission control.

### Limitations

The primary objective of this study was to identify health care organization practices for documenting a patient’s self-reported preference for an interpreter in the EHR, and the adoption level of available standards. We acknowledge that quantifying the impact of standardized interpreter needed data on health outcomes and patient experiences is important; however, this was out of the scope of our project. Future research on the adoption of the interpreter needed data element will help elucidate the impact of standardized data collection practices to address health disparities and improve patient outcomes.

### Conclusion

Improving the quality of services provided to patients with NELP requires health care organizations to systematically collect information from patients regarding their language preferences and to use these data to plan for and offer communication and language assistance services. Despite the presence of common practices for the collection, documentation, and use of data on patient interpretation needs, the lack of standardization limits the exchange of this information among health systems. The use of standard terminologies for interpreter needed data offers one mechanism to facilitate the consistent use of the data within and across health care organizations, resulting in quality improvement [36]. As the US demographics change, there is the potential for unmet patient needs to become more glaring. Standardized data collection and EHR documentation can streamline the use of these data and may help to reduce health disparities.

## Abbreviations

CMS: Centers for Medicare & Medicaid Services
DEL: Data Element Library
EHR: electronic health record
FHIR: Fast Healthcare Interoperability Resources
HL7: Health Level 7
LOINC: Logical Observation Identifiers Names and Codes
NELP: non-English language preference
PAC: postacute care
SNOMED-CT: Systematized Medical Nomenclature for Medicine-Clinical Terminology
USCDI: United States Core Data for Interoperability
